# Burnout and use of HIV services among health care workers in Lusaka District, Zambia: a cross-sectional study

**DOI:** 10.1186/1478-4491-7-55

**Published:** 2009-07-13

**Authors:** Gina R Kruse, Bushimbwa Tambatamba Chapula, Scott Ikeda, Mavis Nkhoma, Nicole Quiterio, Debra Pankratz, Kaluba Mataka, Benjamin H Chi, Virginia Bond, Stewart E Reid

**Affiliations:** 1 Centre for Infectious Disease Research in Zambia (CIDRZ), Lusaka, Zambia; 2 Department of Medicine, Massachusetts General Hospital, Boston, Massachusetts, USA; 3 Lusaka District Health Management Team, Zambian Ministry of Health, Lusaka, Zambia; 4 Department of Family and Social Medicine, Montefiore Medical Center, Bronx, New York, USA; 5 Department of Pediatrics, The Bristol Myers Squibb Children's Hospital, Robert Wood Johnson University Hospital, New Brunswick, New Jersey, USA; 6 Schools of Medicine and Public Health, HIV/AIDS Research in Africa, Department of Obstetrics and Gynecology, University of Alabama at Birmingham, Birmingham, Alabama, USA; 7 ZAMBART Project, Ridgeway Campus, Ridgeway, Lusaka, Zambia; 8 Health Policy Unit, Department of Public Health and Policy, London School of Hygiene and Tropical Medicine, London, UK; 9 School of Medicine, Division of Infectious Disease, University of Alabama at Birmingham, Birmingham, Alabama, USA

## Abstract

**Background:**

Well-documented shortages of health care workers in sub-Saharan Africa are exacerbated by the increased human resource demands of rapidly expanding HIV care and treatment programmes. The successful continuation of existing programmes is threatened by health care worker burnout and HIV-related illness.

**Methods:**

From March to June 2007, we studied occupational burnout and utilization of HIV services among health providers in the Lusaka public health sector. Providers from 13 public clinics were given a 36-item, self-administered questionnaire and invited for focus group discussions and key-informant interviews.

**Results:**

Some 483 active clinical staff completed the questionnaire (84% response rate), 50 staff participated in six focus groups, and four individuals gave interviews. Focus group participants described burnout as feeling overworked, stressed and tired. In the survey, 51% reported occupational burnout. Risk factors were having another job (RR 1.4 95% CI 1.2–1.6) and knowing a co-worker who left in the last year (RR 1.6 95% CI 1.3–2.2). Reasons for co-worker attrition included: better pay (40%), feeling overworked or stressed (21%), moving away (16%), death (8%) and illness (5%). When asked about HIV testing, 370 of 456 (81%) reported having tested; 240 (50%) tested in the last year. In contrast, discussion groups perceived low testing rates. Both discussion groups and survey respondents identified confidentiality as the prime reason for not undergoing HIV testing.

**Conclusion:**

In Lusaka primary care clinics, overwork, illness and death were common reasons for attrition. Programmes to improve access, acceptability and confidentiality of health care services for clinical providers and to reduce workplace stress could substantially affect workforce stability.

## Background

Over the past decade, access to antiretroviral therapy (ART) has grown at an unprecedented rate in sub-Saharan Africa, with tremendous health gains observed among those utilizing these services [1-3]. As programmes have expanded, availability of trained health personnel has become an important limiting factor in the provision of services [4-6].

Nowhere is the shortage more noticeable than in sub-Saharan Africa, where patient: provider ratios fall significantly below World Health Organization recommendations. In 2006, for example, an estimated 2.3 health care workers served every 1000 people in the region. This figure is below the worldwide average of 9.3 per 1000 and below the level defined as a critical shortage: 2.5 per 1000, at which populations fail to achieve 80% coverage of services such as deliveries and vaccinations [7].

Numerous approaches have been implemented to compensate for the shortage. These include task shifting [8,9], employment of lay health workers [10,11] and use of technology (e.g. mobile phones or the Internet) to facilitate care and consultation [11,12]. Few strategies, however, have focused on maintaining the well-being of existing workers [13]. It is crucial that this human resource foundation be kept intact if service provision is to continue in these resource-constrained settings.

In settings with high HIV prevalence, related morbidity and mortality are responsible for health provider absenteeism and attrition. In Zambia, mortality rates are high and before ART was available, death was a common cause of attrition among district health care workers [14,15]. A recent South African survey measured HIV prevalence among health care workers at 12% [16]. In Malawi, the death rate among health care workers was 2%; the most common causes were TB and other chronic illness attributable to AIDS [17].

Perhaps more overlooked is the issue of occupational burnout among health personnel, a phenomenon characterized by exhaustion, depersonalization and inefficacy [18]. The emotional intensity of caring for HIV-infected patients, along with the high patient volumes seen at many ART centres, may place health providers at particular risk for burnout [19]. The consequences can be severe and include exhaustion, reduced productivity, decreased empathy for patients, absenteeism and desire to search out other occupations [20,21].

## Methods

We designed a two-part study to describe occupational burnout and utilization of HIV services among providers in the primary care centres of the Lusaka, Zambia, public health sector, where services for HIV care and treatment have rapidly expanded since 2004 [3]. We recruited physicians, clinical officers (the equivalent of physician assistants in the United States and Europe), nurses, midwives and pharmacy staff employed at government clinics. Thirteen sites were chosen, all providing long-term HIV care and treatment. At each facility, other primary care services are provided, including general outpatient care, antenatal services, child health services and tuberculosis treatment. Characteristics of each facility and catchment size are shown in Table 1.

**Table 1:** District health facilities in Lusaka, Zambia, that served as sites for this study, March 2007 to July 2007

**Clinic**	**Clinic catchment size**	**Staff per 10 000 patients in catchment area**	**Number of active staff during study period**	**Number of survey respondents (%)**
Bauleni	69 899	6.29	44	26 (59%)

Chawama	117 083	4.10	48	48 (100%)

Chelstone	93 065	6.88	64	57 (89%)

Chilenje	98 881	4.65	46	46 (100%)

Chipata	140 464	2.78	39	39 (100%)

George	131 774	3.04	40	35 (88%)

Kabwata	80 212	3.74	30	30 (100%)

Kalingalinga	62 566	8.95	56	25 (44%)

Kamwala	97 191	4.53	44	33 (75%)

Kanyama	139 597	4.01	56	43 (76%)

Matero Main	98 650	2.33	23	20 (86%)

Matero Ref	106 160	4.14	44	42 (95%)

Mtendere	73 565	5.30	39	39 (100%)

Overall	1 309 107	4.38	573	483 (84%)

This study included qualitative and quantitative study components. Our qualitative methods consisted of six focus group discussions and four key-informant interviews. One group session was held for each of the following: midwives, physicians, clinical officers and pharmacy staff. Two groups of nurses were convened, as they comprise the vast majority of health care workers. Participants were stratified according to clinic of employment and professional cadre, and then randomly selected. Overall, 13 invitations were sent out for each group (i.e. one representative per clinic).

Discussions were held in a private conference room at a local nongovernmental organization and each lasted approximately 90 minutes. Trained study staff served as facilitators; written notes and tape recordings were transcribed.

Key informants were identified by physicians based at the study clinics and were recruited to participate in one-on-one interviews. The four interviews included two providers living with HIV, one HIV-negative and one with unknown HIV status. Each lasted approximately 60 minutes.

Data were analyzed by manually compiling common themes and matrices and discussing these data between investigators, study staff and representatives from the Lusaka District Health Management Team.

In parallel, we surveyed active health care providers across the 13 clinics to quantify their perceptions of occupational burnout and HIV service utilization. All staff reporting to work over a three-week window were asked by their supervising nurse managers to complete a 36-question survey. Each questionnaire had a statement of consent attached; completion of this consent was necessary for inclusion in the analysis. Drop boxes were provided so that participants could return their questionnaires anonymously.

In the survey, prevalence of occupational burnout was based on a single question that has been validated against a full occupational burnout scale [22]. Respondents were asked to quantify their level of burnout from a five-item scale: (1) "I have no symptoms of burnout"; (2) "Sometimes I am under stress, but I don't feel burned out"; (3) "I am definitely burned out and have occasional symptoms of burnout"; (4) "The symptoms of burnout I'm experiencing won't go away"; and (5) "I feel completely burned out and I am at the point where I need to make some changes or seek some sort of help". If respondents selected (3), (4), or (5) from the scale, they were categorized as having occupational burnout. We also asked numerous supporting questions to better understand types of burnout in this population.

To determine utilization of HIV services, we relied primarily on use of HIV testing services over the past 12 months. Information regarding demographic characteristics, employment history and HIV knowledge and perceptions was also collected.

In our statistical analysis, we calculated unadjusted and adjusted relative risks (RR) with 95% confidence intervals (95% CI) to identify predictors of occupational burnout and HIV service utilization [23]. We adjusted for individual and clinic-level variation in hierarchical logistic models by means of the SAS GLIMMIX Procedure [24]. Covariates included demographics and other independent variables associated with the outcome at p < 0.10 in unadjusted models. All analyses were performed with SAS version 9.1.3 for Windows (Cary, North Carolina, United States of America).

All participants provided written informed consent to participate. This study was approved by the University of Zambia Research Ethics Committee and the University of Alabama at Birmingham Institutional Review Board.

## Results

In May 2007, 78 individuals were invited for focus group discussions. Fifty (64%) participated: 18 nurses, eight midwives, nine clinical officers, five medical officers and 10 pharmacy staff. Key informants comprised two registered nurses, one nurse manager and one clinical officer.

From March to July 2007, 483 of 573 active health care providers in the 13 clinics completed the study questionnaire (84% response rate). Demographic characteristics are shown in Table 2. The vast majority was female (87%) and the overall median age was 37 (IQR: 31 to 45 years). The median time spent in the district service was 10 years (IQR: 4 to 17 years).

**Table 2:** Demographic and employment characteristics of survey participants (N = 483)

**Variables**	**N**	**n (%)**
Provider type	463	

Physician		7 (1.5%)

Clinical officer		50 (10.8%)

Nurse		234 (50.5%)

Midwife		129 (27.9%)

Pharmacy technician		19 (4.1%)

Other type		24 (5.2%)

Age in years, median (IQR)	451	37 (31–45)

Female	478	414 (86.6%)

Years in present job position, median (IQR)	451	10 (4–17)

Department	454	

Maternal and child health		76 (16.7%)

Outpatient		182 (40.1%)

ART clinic		49 (10.8%)

Inpatient		50 (11.0%)

Labor ward		64 (14.1%)

Other		33 (7.3%)

Marital status	479	

Married		332 (69.3%)

Widowed		64 (13.4%)

Divorced		18 (3.8%)

Single		65 (13.6%)

### Occupational burnout

In our qualitative work, participating health providers related occupational burnout to feelings of being overworked, stressed and tired. Key informants characterized occupational burnout as having low energy levels, being irritable, providing poor treatment, acting rude towards patients, being more prone to mistakes and getting physically sick. Focus group participants attributed their burnout to poor working conditions. They noted long hours and heavy workload: clinical officers reported seeing more than 100 patients in a day.

Low salaries and benefits were a consistent source of frustration. Participants found their salary difficult to survive on and many took on additional jobs, which further exacerbated feelings of burnout. One key informant reported owning a small shop in addition to working nights at the general clinic and mornings at the ART clinic.

These feelings led many government workers to seek "greener pastures," such as private facilities, nongovernmental organizations or other countries where conditions were perceived to be better. It was noted that most health care workers stayed only one to three years before moving on. It was noted that when people leave, more work falls on those remaining.

In the quantitative survey, occupational burnout was noted across numerous metrics. Approximately half of respondents met our definition for occupational burnout. Nearly one quarter of respondents reported feeling too burnt-out to go to work at least once per week. When asked why their co-workers left their positions, the most common reasons were better pay (40%) and because they felt constantly overworked or stressed in their current position (21%), a manifestation of occupational burnout (Table 3). In hierarchical models, significant risk factors for occupational burnout are shown in Table 4.

**Table 3:** Survey respondent experiences with occupational burnout

**Questionnaire Item**	**N**	**n (%)**
Using your own definition of 'burnout', please circle one of the following:	422	

I have no symptoms of burnout.		29 (6.9%)

Sometimes I am under stress, but I don't feel burned out.		177 (42.0%)

I am definitely burning out and have occasional symptoms of burnout.		98 (23.3%)

The symptoms of burnout that I'm experiencing won't go away.		18 (4.3%)

I feel completely burned out. I am at the point where I need to make some changes or seek some sort of help.		99 (23.5%)

I am so burned out that I cannot manage to go for work.	427	

Never		134 (31.4%)

A few times a year		101 (23.7%)

Once a month		52 (12.2%)

Once a week		66 (15.5%)

Every Day		45 (10.5%)

I have become harsh towards my patients.	437	

Never		201 (46.0%)

A few times a year		123 (28.2%)

Once a month		17 (3.9%)

Once a week		30 (6.9%)

Every Day		17 (3.9%)

I do not feel I can sympathize with my clients	397	

Never		229 (57.7%)

A few times a year		58 (14.6%)

Once a month		13 (3.3%)

Once a week		12 (3.0%)

Every Day		25 (6.3%)

How many health care workers in your department have left their position in the last 2 years? median (IQR)	383	2.0 (1.0–4.0)

Why did they leave? (more than one answer possible)		

Better pay		431 (40.2%)

Constantly overstressed: a type of burnout		94 (8.8%)

Constantly overworked: a type of burnout		131 (12.2%)

Moving or leaving the area		175 (16.3%)

Died		83 (7.7%)

Personal health reasons		57 (5.3%)

Do you work at another job to earn extra income?	458	161 (35.2%)

I work at a private health facility		124 (77.0%)

I work at a non-health facility		7 (4.3%)

I am self-employed		32 (19.9%)

How much leave have you taken in the last 12 months? (mean ± SD)	389	28.7 ± 34.8

**Table 4:** Provider-level predictors for reporting occupational burnout

	**Burned-out** **n (%)**	**Not burned-out** **n (%)**	**Crude relative risk** **(95% CI)**	**Adjusted relative risk** (95% CI) *
Male	16 (29.6%)	38 (70.4%)	Ref	Ref

Female	197 (54.4%)	165 (45.6%)	1.8 (1.3–2.9)	2.0 (1.1–2.7)

Age > 45 years	39 (43.3%)	51 (56.7%)	Ref	Ref

Age 36–45 years	71 (55.5%)	57 (45.5%)	1.3 (1.0–1.7)	1.5 (1.1–1.9)

Age 26–35 years	75 (52.5%)	68 (47.5%)	1.2 (0.9–1.6)	1.6 (1.0–2.0)

Age 16–25 years	8 (47.1%)	9 (52.9%)	1.1 (0.6–1.7)	1.8 (0.9–2.2)

Work another job	91 (64.5%)	50 (35.5%)	1.4 (1.2–1.6)	1.4 (1.1–1.6)

Know a co-worker who left	176 (56.8%)	134 (43.2%)	1.6 (1.3–2.2)	1.6 (1.2–2.0)

Worry about acquiring HIV at work	189 (54.5%)	158 (45.5%)	1.5 (1.1–2.2)	1.3 (0.7–1.8)

* Adjusted for gender, age, marital status, general health, job title, department, time in district service, working other jobs, knowing a co-worker who left, and worry about acquiring HIV at work.

### Utilization of HIV services

In qualitative discussions, focus group participants believed that few health care workers had been tested for HIV. When asked to try to quantify this, participants estimated that between 25% and 50% of their colleagues were aware of their HIV status. There was widespread belief that staff members do not seek testing at their clinic of employment, but instead go elsewhere to seek services (e.g. private clinics and nongovernmental organizations). A few participants also reported self-testing for HIV among health care providers, without recommended pre- and post-test counseling.

According to focus group participants, the main reason health providers fail to undergo HIV testing was concern over confidentiality. Every focus group acknowledged a perceived or actual lack of confidentiality by co-workers. One key informant overheard nurses discussing the circumstances surrounding her decision to seek HIV testing (i.e. her husband's illness). A medical officer observed: "In the clinic the whole staff are confidential with a patient's history, but when it comes to a clinical officer, the whole staff would be interested." Focus group participants also reported that if a health care provider were known to be living with HIV, he or she would lose the confidence of patients and his or her future employment prospects would be compromised.

Focus group participants were afraid of becoming infected with HIV at work through activities such as injections, blood collection, intravenous infusions and deliveries. Despite this concern, many felt obligated to put themselves at risk during procedures for the sake of the patient. As one nurse explained: "If you do it, you risk your life, and if you do not, the patient dies." However, every discussion group believed most HIV-infected health care workers acquired the disease through their personal life rather than occupational exposure.

Focus group participants reported significant stigma associated with HIV. In one case, a staff member with known HIV infection used another staff member's cup. When the owner of the cup discovered this, she broke it rather than reusing it. Participants believed that stigmatization contributes to staff avoiding or delaying HIV testing.

Despite these examples, many insisted that stigma was decreasing and supported disclosure of one's status as the best way to cope with a diagnosis of HIV. Participants reported that colleagues who were open with their HIV-positive status were treated equally. However, many agreed that HIV-infected staff members were more likely to be perceived as ill and therefore given lighter work assignments. The subsequent increase in workload for others was sometimes resented.

In our survey of health providers, 52% reported undergoing HIV testing in the last 12 months. Of these, more than half (54%) reported having been tested in their clinic of employment (Additional file 1). When respondents who had not undergone HIV testing were asked why not, confidentiality was cited as the chief concern among 28 of 60 (47%) respondents. Most respondents (87%) worried about becoming infected with HIV during their work as a health care provider. In contrast to our focus groups, more than one third of our respondents believed that most HIV-positive health care workers were infected at work. There were fears regarding unlikely modes of transmission, including through sweat (14%) and saliva (25%).

Most survey respondents (87%) reporting knowing at least one health worker infected with HIV, usually after the colleague directly disclosed his or her HIV status. However, other modes of disclosure were reported that involved breaches of confidentiality (Additional file 1). One hundred ninety-five of 474 (41%) had personally witnessed health providers gossiping about a patient's HIV status. In multivariate models, work in the ART clinic (RR = 1.4, 95%CI = 1.0 – 1.6) and worry about becoming infected with HIV during their work (RR = 1.8, 95%CI 1.2 – 2.2) were significantly associated with HIV testing (Table 5).

**Table 5:** Provider-level predictors for undergoing an HIV test over the 12 months

	**Tested** **n (%)**	**Not tested** **n (%)**	**Crude relative risk** **(95% CI)**	**Adjusted relative risk** (95% CI) *
Male	30 (50.0%)	30 (50.0%)	Ref	Ref

Female	207 (52.0%)	191 (48.0%)	1.0 (0.8–1.4)	1.1 (0.7–1.5)

	57 (58.8%)	40 (41.2%)	Ref	Ref

Age 36–45 years	49 (36.3%)	86 (63.7%)	0.6 (0.5–0.8)	0.6 (0.4–0.9)

Age 26–35 years	96 (57.1%)	72 (42.9%)	1.0 (0.8–1.2)	1.0 (0.7–1.3)

Age 16–25 years	14 (82.4%)	3 (17.7%)	1.4 (1.0–1.7)	1.5 (1.0–1.7)

Age >45 years	91 (52.9%)	81 (47.1%)	Ref	Ref

ART clinic	30 (63.8%)	17 (36.2%)	1.2 (0.9–1.5)	1.4 (1.0–1.6)

Inpatient department	21 (47.7%)	23 (52.3%)	0.9 (0.6–1.2)	0.9 (0.5–1.3)

Maternal child health	42 (56.0%)	33 (44.0%)	1.1 (0.8–1.3)	1.1 (0.6–1.5)

Labor ward	26 (40.6%)	38 (59.4%)	0.8 (0.5–1.0)	1.0 (0.5–1.5)

Other department	16 (50.0%)	16 (50.0%)	0.9 (0.6–1.3)	1.4 (0.7–1.7)

Worry about acquiring HIV at work	213 (54.8%)	176 (45.2%)	1.5 (1.1–2.2)	1.8 (1.2–2.2)

Know coworker has HIV because he/she told me	158 (54.3%)	133 (45.7%)	1.1 (0.9–1.4)	1.2 (1.0–1.4)

* Adjusted for gender, age, marital status, general health, job title, department, worry about acquiring HIV at work, and knowing a coworker has HIV because he/she disclosed to them.

## Discussion

In this study, we found a high prevalence of occupational burnout among district health staff. Over half met our screening definition, with most reporting numerous symptoms. Conditions of service were the most commonly cited causes of occupational burnout and attrition. Only half of our respondents reported having been tested for HIV in the past 12 months, despite the widespread availability of such services in the clinic of employment and "stand-alone" testing sites in the community. Stigma remained a significant reason why health care workers avoided HIV testing; it continued to serve as a barrier to widespread HIV testing among providers.

A strength of this study was its combination of qualitative and quantitative methods. This allowed us to "triangulate" data from multiple sources and provide comprehensive descriptions of our topics of interest. Our primary limitation is external validity. We do not know the degree to which our findings are specific to the primary care clinics of the Lusaka public health sector; however, we believe the themes are applicable to other urban African settings. We are also aware of potential reporting bias, particularly in descriptions of occupational burnout. Focus group participants were familiar with the phenomenon of burnout, likely due to prior district-sponsored workshops on the topic. In addition, all participants were informed that this study was designed to inform district policy. These conditions may have provided incentives for staff to overestimate burnout prevalence.

Occupational burnout was prevalent in this population of government health workers in these Lusaka primary care facilities. One significant predictor for occupational burnout was knowing a co-worker who had left government service. Those who remain are left with more stressful conditions, putting them at risk for burnout. This is a cycle that deserves further investigation, given its potential for exacerbating the burnout phenomenon in settings where health staff are limited. Furthermore, patients may suffer in this process, as both occupational burnout and low staffing levels have been associated with a decline in quality of care [21,25]. Measuring the effect of health care provider burnout on patient outcomes is an important area for future research, with powerful implications for maintaining HIV care and treatment programmes.

More than half of our survey respondents had been tested for HIV over the past 12 months. Although this figure is higher than from other reports in the region [26,27], it was below our expectations. Nearly one quarter said they had "not had the opportunity to test" for HIV, despite the availability of services across numerous different testing venues (e.g. public clinics, private facilities and stand-alone testing centres).

Participants in both study components conveyed worry over accidental disclosure of HIV infection. Ironically, this fear of disclosure has made utilization of HIV services more difficult for these ministry employees, since their familiarity with clinic staff may make them vulnerable to breaches in confidentiality, speculation and gossip. Confidentiality and gossip have been recognized as deterrents to HIV testing among general populations [28]. Clinic staff working to provide ART were more likely to seek testing for HIV, when compared to those from other departments. This may be related to reduced stigma within this department or greater concern over HIV transmission from infected patients.

The role of occupational exposure in HIV acquisition was inconsistent between our qualitative and quantitative components. Focus groups attributed HIV infection among providers to non-work-related risk factors (75% to 90%) while about 40% of survey respondents believed that occupational exposure was the major route of transmission. This discrepancy may be partially attributed to misconceptions by survey respondents that HIV could be transmitted by sweat or saliva. If health care workers perceive themselves to be at high risk for HIV infection based on these misconceptions, it could adversely affect their likelihood of seeking testing. A study among Zambian women found that women reporting high personal risk for infection were less likely to accept testing at antenatal facilities [29].

Despite their roles in providing care for HIV-infected patients, health staff may have persistent misinformation and misconceptions about the disease [30]. In our survey, for example, nearly all respondents stated that HIV-infected individuals should be treated with the same respect as those who are not infected. At the same time, large proportions blamed promiscuous men or prostitutes for spreading the epidemic. While the opposing nature of these views may be subtle, they do speak to underlying and persistent stigma – even among health providers.

The overall goal of this study was to further inform district policy regarding workplace conditions. These results have been formally presented to clinic nurse managers and the Lusaka District Health Management Team. The ensuing discussion resulted in numerous recommendations that are currently being considered at the district level. To combat occupational burnout, district staff suggested workshops to teach individuals to identify burnout and develop coping mechanisms.

To improve utilization of HIV-related health services, staff emphasized the need for multiple options, since one approach was unlikely to meet the needs of all. Possibilities included: (1) parallel systems within clinics where staff could get care from a trusted provider; (2) a central HIV/AIDS clinic for staff; or (3) a central comprehensive health care clinic for staff, offering both ART and general care.

Peer support groups for HIV-infected health care providers were functioning in one clinic; this was seen as a success and it was suggested that other clinics implement this strategy. Ongoing initiatives to combat confidentiality breaches and HIV-associated stigma were promoted. Formal workplace HIV policies were recommended to address the unique challenges of confidentiality and stigma faced by health care workers in gaining access to HIV services.

## Conclusion

As new initiatives are implemented to increase health personnel capacity in sub-Saharan Africa, existing health providers must not be overlooked. The burden of providing HIV services to large numbers of extremely ill patients is substantial and may lead to high levels of occupational burnout. Working conditions should be regularly evaluated and where possible improved, to prevent attrition related to occupational burnout. Initiatives must also focus on improving uptake of HIV testing, care and treatment services among health providers. This may require investment in locally appropriate clinical care options that will ensure confidentiality and formal workplace policies to protect those who disclose their status.

## Competing interests

The authors declare that they have no competing interests.

## Authors' contributions

GRK contributed to the initial study concept, protocol design, oversight, implementation, data analysis and initial drafting of the manuscript. BT contributed study oversight, implementation and critical edits to the manuscript. SI contributed to the initial study concept, protocol design, oversight and implementation and critical edits to the manuscript. MN contributed to oversight of data collection and implementation. NQ contributed to oversight and implementation and critical edits to the manuscript. DP contributed to oversight of data collection, data entry, implementation and editing the final version. KM contributed to oversight of data collection, data entry and analysis. BHC contributed to data interpretation and initial drafting and critical edits to the manuscript. VB contributed to the study design, review of the analysis and critical edits to the manuscript. SER contributed to the initial study concept, protocol design, oversight and implementation and critical edits to the manuscript. All authors read and approved the final manuscript.

## Supplementary Material

Additional file 1 **Respondent experiences with HIV service use and stigma**. Supplementary table outlining the HIV service use and stigma experiences of respondents. Click here for file
