# Supplementary material for: Burnout and use of HIV services among health care workers in Lusaka District, Zambia: a cross-sectional study
Source: Hum Resour Health. 2009 Jul 13;7:55. doi: 10.1186/1478-4491-7-55 (PMC2714832; doi:10.1186/1478-4491-7-55)
Supplement: Additional file 1 — Respondent experiences with HIV service use and stigma. Supplementary table outlining the HIV service use and stigma experiences of respondents. [file 1478-4491-7-55-S1.doc]

### Respondent experiences with HIV service use and stigma

| **Questionnaire Item** | **N** | **n (%)** |
| --- | --- | --- |
| Have you ever been tested for HIV? | 461 | 375 (81.3%) |
| How long ago was your last HIV test? | 461 |  |
| Tested in the last 12 months |  | 240 (52.1%) |
| Where were you tested for HIV? | 367 |  |
| In this clinic |  | 199 (54.2%) |
| Another district clinic |  | 68 (18.5%) |
| Private health care worker clinic |  | 35 (9.5%) |
| Other location |  | 54 (14.7%) |
| Why have you never been tested? (more than one answer possible) | | |
| I have not had the opportunity to be tested | 54 | 12 (22.2%) |
| I do not feel I am at risk | 59 | 11 (18.6%) |
| I am afraid that my colleagues will find out | 55 | 6 (10.9%) |
| I am afraid that people will talk about me | 58 | 11 (19.0%) |
| I cannot be assured of confidentiality | 60 | 28 (46.7%) |
| Do you worry about getting HIV during your work as a health care provider? | 462 | 399 (86.4%) |
| In response to the following situations, please indicate whether or not you can get HIV from the following activities: | | |
| Touching the sweat of a person with HIV or AIDS. | 445 |  |
| Yes |  | 64 (14.4%) |
| No |  | 357 (80.2%) |
| Do not know |  | 24 (5.4%) |
| Touching the saliva of a person with HIV or AIDS. | 444 |  |
| Yes |  | 112 (25.2%) |
| No |  | 307 (69.1%) |
| Do not know |  | 25 (5.6%) |
| Giving an injection to a person with HIV or AIDS. | 435 |  |
| Yes |  | 120 (27.6%) |
| No |  | 312 (71.7%) |
| Do not know |  | 3 (0.7%) |
| Inserting an intrauterine device for a person with HIV or AIDS. | 424 |  |
| Yes |  | 115 (27.1%) |
| No |  | 292 (68.9%) |
| Do not know |  | 17 (4.0%) |
| Caring for a person with HIV or AIDS. | 442 |  |
| Yes |  | 132 (29.9%) |
| No |  | 305 (69.0%) |
| Do not know |  | 5 (1.1%) |
| Dressing the wounds of a person with HIV or AIDS. | 431 |  |
| Yes |  | 171 (39.7%) |
| No |  | 255 (59.2%) |
| Do not know |  | 5 (1.2%) |
| Putting up a drip on someone who is showing signs of AIDS. | 428 |  |
| Yes |  | 121 (28.3%) |
| No |  | 303 (70.8%) |
| Do not know |  | 4 (0.9%) |
| Do you agree or disagree with the following statements: (proportion who agree listed below) | | |
| HIV is apunishment from God. | 468 | 22 (4.7%) |
| HIV is punishment for bad or immoral behavior. | 465 | 57 (12.3%) |
| People with HIV should be ashamed of themselves. | 469 | 12 (2.6%) |
| Promiscuous men are the ones that spread HIV. | 458 | 264 (57.6%) |
| It is the women prostitutes who spread HIV in our community, | 469 | 225 (48.0%) |
| I would feel ashamed if I was infected with HIV. | 466 | 41 (8.8%) |
| I would feel ashamed if someone in my family was infected with HIV. | 470 | 26 (5.5%) |
| People with HIV should be treated with the same respect as everyone else. | 471 | 458 (97.2%) |
| Do you know of a health care worker/colleague who has HIV infection? | 473 | 411 (86.9%) |
| How did you know that he/she has HIV infection? | 411 |  |
| The infected person told me her/himself. |  | 300 (73.0%) |
| Family member of infected person told me. |  | 4 (1.0%) |
| Community member told me. |  | 2 (0.5%) |
| General gossip/rumors. |  | 38 (9.2%) |
| From a health care provider where the person tested. |  | 9 (2.2%) |
| Read from his/her hospital file. |  | 21 (5.1%) |
| I recognized signs or symptoms that may be from HIV. |  | 32 (7.8%) |
| I work in the ART clinic where he/she tested or gets treated |  | 15 (3.6%) |
